# Non-Genotoxic and Environmentally Relevant Lower Molecular Weight Polycyclic Aromatic Hydrocarbons Significantly Increase Tumorigenicity of Benzo[*a*]pyrene in a Lung Two-Stage Mouse Model

**DOI:** 10.3390/toxics12120882

**Published:** 2024-12-02

**Authors:** Alison K. Bauer, Deedee Romo, Finnegan Friday, Kaila Cho, Kalpana Velmurugan, Brad L. Upham

**Affiliations:** 1 Department of Environmental and Occupational Health, Colorado School of Public Health, University of Colorado Anschutz Medical Campus, Aurora, CO 80045, USA; deedee.romo@cuanschutz.edu (D.R.); finnegan.friday@cuanschutz.edu (F.F.); kaila.cho@ucdenver.edu (K.C.); kalpana.velmurugan@cuanschutz.edu (K.V.); 2 Department of Pediatrics and Human Development, Michigan State University, East Lansing, MI 48824, USA; upham@msu.edu

**Keywords:** polycyclic aromatic hydrocarbons, benzo[*a*]pyrene, fluoranthene, 1-methylanthracene, phenanthrene, tumor promoter, lung, mice

## Abstract

The World Health Organization has classified air pollution as a carcinogen, and polycyclic aromatic hydrocarbons (PAHs) are major components of air particulates of carcinogenic concern. Thus far, most studies focused on genotoxic high molecular weight PAHs; however, recent studies indicate potential carcinogenicity of the non-genotoxic lower molecular weight PAHs (LMW PAHs) that are found in indoor and outdoor air pollution as well as secondhand cigarette smoke. We hypothesize that LMW PAHs contribute to the promotion stage of cancer when combined with benzo[*a*]pyrene (B[*a*]P), a legacy PAH. We specifically determined the effects of an LMW PAH mixture containing 1-methylanthracene (1MeA), fluoranthene (Flthn), and phenanthrene (Phe) combined with B[*a*]P on lung tumor promotion. To test this hypothesis, we used a two-stage, initiation/promotion BALB/ByJ female lung tumor mouse model. The mice were initiated with 3-methylcholanthrene followed by exposures to B[*a*]P, the LMW PAH mixture, and the combination of the LMW PAH mixture plus B[*a*]P, all at 10 mg/kg. The LMW PAHs combined with B[*a*]P significantly increased the promotion and incidence of lung tumors over that of B[*a*]P alone. The LMW PAHs in the absence of B[*a*]P did not significantly promote tumors, indicating strong co-promotional activities. We further assessed the effects of these PAHs on other hallmarks of cancer, namely, bronchoalveolar lavage fluid inflammatory infiltrates, pro-inflammatory transcripts, KC protein content, and mRNA expression of the gap junction (*Gja1*) and epiregulin (*Ereg*) genes. The LMW PAHs increased the biomarkers of inflammation, decreased *Gja1* expression, and increased *Ereg* expression, all consistent with tumor promotion. This study indicates that non-genotoxic LMW PAHs can contribute to the cancer process and warrants further studies to assess the carcinogenic risks of other LMW PAHs.

## 1. Introduction

Lung cancer is the deadliest cancer worldwide, and lung adenocarcinoma (LUAD) is the most common type of lung cancer among both smokers and non-smokers, with causes such as environmental exposures (e.g., air pollution and secondhand cigarette smoke) [1,2]; few if any interventions exist for LUAD [3]. Air pollution, which includes ambient particulate matter (PM) size 2.5 microns (PM_2.5_), is classified as a carcinogen, according to the World Health Organization (WHO) [4]. Recent evidence determined a significant, positive association between exposure to ambient PM_2.5_ and LUAD development in a three-country-level cohort [5]. Polycyclic aromatic hydrocarbons (PAHs) are toxicants of concern that are components of PM_2.5_ from both indoor and outdoor air pollution [6]. PAHs are generated during incomplete combustion of carbon sources, such as gasoline and diesel exhaust [7,8,9,10], coke oven emissions [11], wildfire particulate matter [12,13,14], burn pits [15], and primary and secondhand smoke [16,17]. Workers are exposed to PAHs in many occupations such as asphalt, coal, coke, and the steel industry [18,19,20]. For example, in more recent studies, PAHs were detected in the urine and blood of wildland firefighters [12,13,14] as well as in the lungs of soldiers exposed to burn pits [21]. 

Some PAHs are categorized as carcinogens by agencies such as the international agency for research on cancer (IARC), under the WHO [22]. These are largely the higher molecular weight PAHs (typically >5 ring structures) that are considered carcinogens according to IARC. For example, benzo[*a*]pyrene (B[*a*]P) is classified as a group 1 carcinogen by the IARC [23] and the United States Environmental Protection Agency (EPA) [24]. However, many other PAHs, such as lower molecular weight PAHs (LMW; typically ≤4 ring structures) are *unclassifiable* (group 3) as to their carcinogenic potential, including methylated anthracenes, phenanthrene, and fluoranthene, due to a lack of supportive evidence. Two exceptions exist; naphthalene is a group 1 carcinogen [25], and anthracene was recently reclassified as a group 2B carcinogen in 2023 [26]. To address this gap in knowledge, our group has focused on the potential tumorigenicity of lower molecular weight PAHs and published seminal papers using in vitro models demonstrating the potential tumorigenicity of LMW PAHs [27,28,29,30,31,32], and in this study we provide, for the first time, in vivo evidence that LMW PAHs contribute to promoting tumors in a lung mouse model.

**Figure 1:**
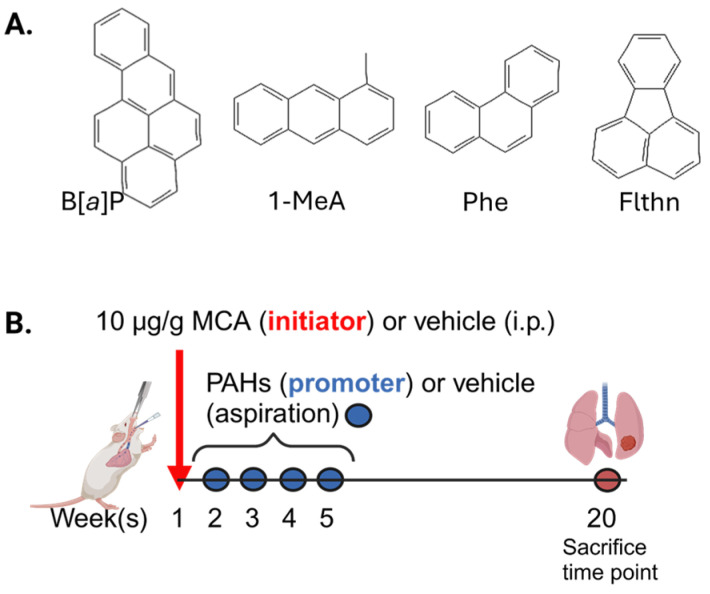
A schematic of the two-stage model to test the novel concept that a non-genotoxic LMW PAH mixture can promote lung tumors. (**A**). Structures of benzo[*a*]pyrene (B[*a*]P), 1-methylanthracene (1-MeA), phenanthrene (Phe), and fluoranthene (Flthn). (**B**). The two-stage model consists of the initiator (3-methylcholanthrene, a high molecular weight PAH) administered by ip. injection dissolved in corn oil (vehicle) on week 1, followed by 4 weekly doses of the PAHs, at a 10 mg/kg total PAH concentration. B[*a*]P and the LMW PAH mixture (1-MeA, Phe, and Flthn) were administered by themselves or in combination by oropharyngeal aspiration (25 μL) directly to the lung all dissolved in tricaprylin (vehicle). These PAHs are all components of wildfire smoke particulates, burn pits, secondhand smoke, among many other potential exposures. Created in BioRender. Bauer, A. (2024) https://BioRender.com/i74r442, Access date (30 October 2024).

More specifically to lung tumors, we previously demonstrated that exposing mouse and human lung epithelial cell lines to B[*a*]P combined with two LMW PAHs, 1-methylanthracene (1-MeA) and fluoranthene (Flthn), induced changes in numerous hallmarks of cancer. These hallmarks included increases in genotoxicity measured by micronuclei, anti-benzo(a)pyrene diol-epoxide-DNA adduct formation, and pro-inflammatory/tumorigenic mediators (tumor-promoting inflammation hallmarks) [27,33,34,35]. The LMW PAH binary mixtures (1-MeA + Flthn) without B[*a*]P also altered inflammatory pathways with increases observed in pro-inflammatory bioactive lipid pathways, such as cytosolic phospholipase A2 (cPLA_2_), cyclooxygenase (COX2), and downstream prostaglandins (PGE_2_, PGD_2_), isoprostanes, and pro-inflammatory cytokines, such as keratinocyte-derived chemokine (KC), IL6, TNF, and IL1B, among others. When combined with B[*a*]P, these LMW PAHs significantly increased *Cox2* mRNA expression compared to either alone. Significant increases were observed in proliferation and MAP kinase pathways linked to proliferation and inflammation (i.e., MAPK ERK1/2 and P38) in response to these LMW PAHs. An additional hallmark evaluated was the evasion of growth suppression tested by measuring gap junction intercellular communication (GJIC) dysfunction. GJIC dysfunction is critical in early-stage cancer and supported by a considerable amount of evidence (see these references [36,37]). GJIC was significantly reduced by LMW PAHs [27,28,30] and further inhibition of GJIC was observed when combined with B[*a*]P [27]. Collectively, these changes in biomarkers representing hallmarks of cancer in response to LMW PAHs provide evidence that these LMW PAHs can contribute to the cancer process. In addition, 1-MeA, Flthn, and another LMW PAH, phenanthrene (Phe) (see Figure 1A), are highly abundant PAHs in the above-mentioned exposures from burn pits [15] and wildfires [12,13,14] to secondhand smoke exposures [16]. In addition to the environmental significance of these selected PAHs, we have conducted many in vitro mechanistic studies using these specific LMW PAHs [27,28,29,30,31,32], thus providing potential mechanistic links between in vitro and in vivo models.

Importantly, few studies have evaluated these LMW PAHs for carcinogenic potential in the context of a primary animal model [38,39,40], and none have assessed these PAHs in the context of a two-stage lung initiation and promotion model. Environmental lung carcinogenesis is a multi-stage process that begins with the initiation, where the precursor cells undergo DNA damaging/genotoxic events, followed by tumor promotion, where key components lead to cellular transformation and, finally, progression [41,42]. Promoters do not result in tumor development without the presence of an initiator, such as 3-methylcholanthrene (MCA). Thus, these unique two-stage models are important because they test whether these PAHs can initiate tumorigenesis or if they can only promote tumors after the initiation stage. *We hypothesize that when the LMW PAH mixture (1MeA, Flthn, and Phe) is combined with B[a]P, lung tumor promotion is observed* (see Figure 1). To evaluate this hypothesis, we analyzed BALB/ByJ mice for tumor development, tumor incidence, bronchoalveolar lavage fluid inflammatory infiltrates, pro-inflammatory genes, KC protein content, immunostaining, and two additional markers for hallmarks of early-stage LUAD: a gap junction gene (*Gja1*) and epiregulin (*Ereg*; an epidermal growth factor receptor (EGFR) ligand).

## 2. Materials and Methods

### 2.1. Two-Stage Initiation Promotion Lung Model

Five-week-old female BALB/ByJ mice were purchased from Jackson Laboratories (Bar Harbor, MN, USA) and acclimated for 1 week prior to entering the study in a pathogen-free facility. This strain has an intermediate sensitivity to the development of lung tumors, specifically LUAD, and has been used previously by our laboratory using vanadium pentoxide or butylated hydroxytoluene as promoters [43,44,45]. The mice were fed irradiated mouse chow (Harlan) and water *ad libitum* and housed in shoebox cages with the humidity and temperature controlled. All animal use was conducted in facilities accredited by the Association for the Assessment and Accreditation of Laboratory Animal Care and approved by the University of Colorado Denver Institutional Animal Care and Use Committee. 

The mice were randomized into groups for the following protocol (see Figure 1B). On week 1, 10 μg/g MCA or corn oil was intraperitoneal (ip.) injected to initiate the mice. MCA induces *Kras* mutations in BALB/ByJ mice similar to those in smokers and is a well-established initiator for lung adenomas and LUADs [46]. One week following initiation, for the next four weeks, once weekly, the mice were oropharyngeal aspirated with 25 μL of PAHs or tricaprylin (vehicle; Sigma-Aldrich, Inc., St. Louis, MO, USA) [47] to determine promotion. The PAHs used for the aspirations were used at a total concentration of 10 mg/kg PAH (equivalent masses), regardless of the mixture. The mixtures were the lower molecular weight (LMW) PAH mixture (1-MeA:Flthn:Phe; equivalent mass at 3.33 mg/kg per PAH), B[*a*]P at 10 mg/kg, or the combination of B[*a*]P and the LMW PAH mixture at 5 mg/kg B[*a*]P and 1.7 mg/kg for each LMW PAH (1MeA:Flthn:Phe) for a total of 10 mg/kg. These doses were used based on previous studies in the lung with B[*a*]P that used 50 mg/kg per week for 4 weeks [47] and Flthn (ip.) at ~175 mg/kg per dose administered 3 times during 2 weeks [38,39,40]. In addition, known concentrations of LMW PAH exposure in secondhand smoke can reach the mg level [16,17]. Thus, we used a robust dose for this first study to determine a mixture effect. The mice were euthanized with Fatal Plus (120 mg/kg; MWI, Boise, ID, USA) 20 weeks following MCA. The mice were used for the tumor endpoints and bronchoalveolar lavage fluid (BALF) analysis, including ELISA, and a subset of the mice were used for molecular analysis (e.g., quantitative RT-PCR).

### 2.2. BALF Analysis

Following termination, whole lungs were lavaged (volume according to body weight) using 1X Hanks Balanced Salt Solution (HBSS) with 0.6 mM ethylenediaminetetraacetic disodium salt (EDTA) to determine changes in cellular infiltrates in the lung, as performed previously [43,44,45]. Briefly, cell differentials were determined following the staining of cytospin slides of BALF cells using HEMA 3 staining solutions (Fisher Scientific, Waltham, MA, USA) per the manufacturer’s protocol. Total protein, a measure of lung hyperpermeability, was measured using the BioRad assay (BioRad, Hercules, CA, USA). The remaining BALF was frozen for the keratinocyte-derived chemokine (KC) enzyme-linked immunosorbent assay (ELISA).

### 2.3. Lung Tumor Endpoints: Multiplicity and Histopathology

Following BALF, the lungs were fixed in 10% neutral buffered formalin (Sigma, St. Louis, MO, USA), and tumors were enumerated using an Olympus SZX7 dissecting microscope (Olympus, Center Valley, PA, USA) in a blinded manner. Tumor multiplicity is the average number of tumors per mouse. Tumor incidence is the number of mice with tumors. Tumors were also sized using a digital caliper (Mitutoyo, Aurora, IL, USA) and calculated using the area formula (0.5236 L1(L2)^2^), where L1 is the long axes and L2 is the short axes of the tumor. The lungs were then processed, embedded in paraffin, and 5 μm lung sections were cut at the CU Anschutz Cancer Center Pathology Shared Resource. Tumor morphology was determined by light microscopy (Olympus BX43) using hemotoxylin and eosin (H&E) sections. Additional 5 μm lung sections were used for immunostaining.

### 2.4. RNA Isolation, cDNA Synthesis, and Quantitative PCR (qRT-PCR)

RNA was isolated using the Macherey–Nagel Nucleospin RNA II kit (Clontech Laboratories, Mountain View, CA, USA) following their kit specifications. One microgram of total RNA was reverse-transcribed to cDNA [28] and amplified with gene-specific primers labeled with Power SYBR Green master mix (Applied Biosystems, Foster City, CA, USA) using a QuantStudio 3 Real-time PCR (Applied Biosystems, Waltham, MA, USA). Samples were normalized to the expression of 18S rRNA using the comparative CT method [28]. Sequences for the primers can be found here: *cPla2 forward (5′*→*3′)* cagatccttatgtggaactt, *cPla2 reverse (5′*→*3′)* aacttccagagacatttcca; *Cd206 forward (5′*→*3′)* atgaggcttctcctgcttct, *Cd206 reverse (5′*→*3′)* tgatctgagaatctgacacc; *Il10 forward (5′*→*3′)* gctcttactgactggcatgag, *Il10 reverse (5′*→*3′)* cgcagctctaggagcatgtg; all others are in previously published works for *Mcp-1* (*Ccl2*), cyclooxygenase (*Cox)2*, gap junction alpha 1 (Gja1), *Il6*, inducible nitric oxide synthase (*iNos)*, arginase (*Arg)1*, epiregulin (*Ereg)*, and 18S [28,43,44,48,49,50].

### 2.5. KC (CXCL1) ELISA

Detection of the chemokine KC was determined by an R and D Duoset ELISA kit for KC (CXCL1) purchased from R&D systems (Minneapolis, MN, USA), following the manufacturer’s instructions [44]. A Tecan Infinite M200 PRO Microplate Reader with Magellan 7.0 Software (Morrisville, NC, USA) was used to read the ELISA at 450 nm.

### 2.6. Immunohistochemistry on Lung Sections

Immunohistochemistry for macrophages (F4/80), neutrophils (PMNs), and COX2 was performed following previous methods with some modifications noted here. F4/80 staining was performed by the CU Cancer Center Pathology Shared Resource with a 1:200 dilution of the F4/80 antibody (Cell Signaling #70076, Danvers, MA, USA). The other markers were performed in our laboratory, similar to previous studies [44,48]. Briefly, sections were re-hydrated, and endogenous peroxidase activity was inhibited with 3% H_2_O_2_ followed by antigen retrieval using 100 mM citrate buffer. For neutrophils, after blocking with 10% rabbit serum in PBS (Vector Laboratories, Burlingame, CA, USA), primary PMN rat antibody (1:350; Santa Cruz, NIMP-R14, #59338, Dallas, TX, USA) was applied and incubated overnight at 4 °C. Vectastain Elite ABC-HRP anti-rat secondary antibody (Vector Laboratories) was applied, and the sections were stained using the Vectastain Elite ABC-HRP kit (Vector Laboratories) with ImmPACT 3,3-diamino-benzidine (DAB) substrate for visualization. For COX2, slides were blocked in 5% goat serum in tris-buffered saline, followed by incubation with primary COX2 rabbit antibody (Cell Signaling, #12282; 1:600) overnight at 4 °C. SignalStain Boost Detection Reagent (HRP, Rabbit #8114, Cell Signaling) was then applied for 30 min followed by SignalStain DAB substrate (Cell Signaling). Gill’s hematoxylin solution (Vector Laboratories) was used as a counterstain for both the PMN and COX2 immunostaining. Images for all immunostaining and histology were performed using an Olympus BX43 microscope with a DP28 camera and Cellsens Entry software ver. 4.2.

### 2.7. Statistical Analysis

GraphPad Prism (Version 10, La Jolla, CA, USA) was used for all graphs and statistical evaluations; *p* < 0.05 was considered statistically significant. All data are presented as group mean ± SEM. ANOVA was used for all analyses, followed by Tukey’s multiple comparison test for *a posteriori* comparison of means. 

## 3. Results

### 3.1. Tumor Promotion Observed in Response to the Combined PAHs

**Figure 2:**
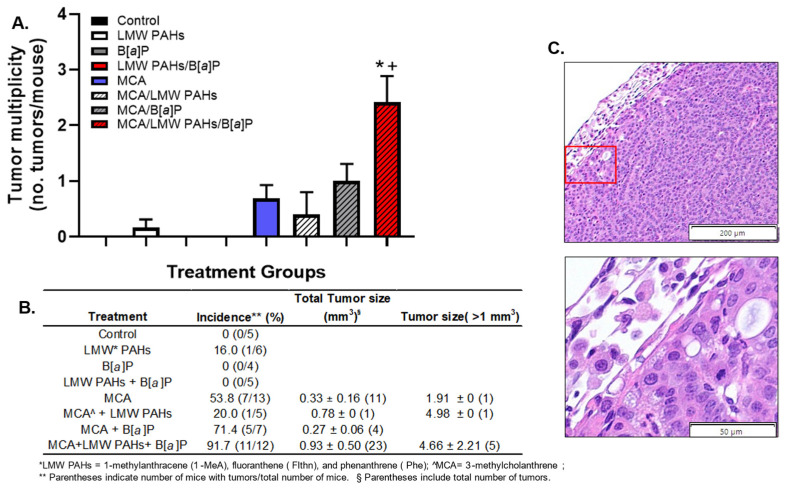
Tumor multiplicity significantly increases only in initiated BALB/ByJ mice with both the LMW PAH mixture and B[*a*]P combined. (**A**) Tumor multiplicity in response to a novel two-stage lung carcinogenesis model. Mean ± SEM presented. Control, n = 5; LMW PAH mixture, n = 6; B[*a*]P, n = 4; LMW PAH mixture + B[*a*]P, n = 5; MCA, n = 13; MCA + LMW PAH mixture, n = 5; MCA + B[*a*]P, n = 7; MCA +LMW PAH mixture + B[*a*]P, n = 12. (**B**) Table of tumor incidence (the number of mice with tumors for all treatment groups) and tumor size. Vehicle control for MCA is corn oil and vehicle control for all other PAHs is tricaprylin; LMW PAH mixture of 1-methylanthracene (1-MeA), fluoranthene (Flthn), and phenanthrene (Phe) in an equivalent mass concentration (1:1:1); benzo[*a*]pyrene, B[*a*]P; 3-methylcholanthrene, MCA. All PAHs tested as promoters are at the 10 mg/kg total PAH concentration. For example, the 1MeA:Flthn:Phe mixture is a 1:1:1 mass ratio for this PAH mixture. For the LMW PAH mixture only, the concentrations are all at 3.33 mg/kg. *, *p* < 0.05 from control; + *p* < 0.05 from MCA and all other groups. (**C**) Representative image from an H & E-stained LUAD from a mouse treated with MCA plus the combination of the LMW PAH mixture and B[*a*]P. Magnification at 10X is reflected by the 200 μm bar and at 40X is reflected by the 50 μm bar. The red box indicates the location of the 40X magnification.

We evaluated the mice for tumor multiplicity 20 weeks following the initiation using MCA and observed significant increases in tumor numbers/mouse in the mice exposed to the combination of MCA with both the LMW PAH mixture and B[*a*]P (Figure 2A; MCA/LMW PAH mixture/B[*a*]P). In fact, tumor multiplicity was not above one tumor per mouse in any group except the combination of MCA with both the LMW PAH mixture and B[*a*]P. Tumor incidence, the number of mice with a lung tumor, also differed between groups (Figure 2B), with only one group with any tumors without MCA initiation (i.e., the LMW PAH mixture). For anenvironmental toxicant exposure, this difference in incidence among those initiated groups is intriguing given the ~20% difference in incidence between the MCA/B[*a*]P group and the MCA/LMW PAH mixture/B[*a*]P group. The image in Figure 2C represents one of the tumors observed in the combined MCA/LMW PAH mixture/B[*a*]P group with a mixed morphology of both solid and papillary features in an LUAD. The tumors observed in the MCA and MCA/B[*a*]P group were 100% a solid morphology; however, in the MCA/LMW PAH mixture/B[*a*]P group, the tumors were 63.6% solid and 36.4% a mixed papillary/solid morphology. All tumors observed were adenomas except in the MCA/LMW PAH mixture/B[*a*]P group with 18.2% LUAD observed and 81.8% adenomas. Additional H & E images are found in Figure S1, demonstrating the increase in cellularity observed as well as the thickening of the airways and airspaces, specifically in the LMW PAH mixture groups (Figure S1B,F,D,H). The overall tumor sizes did not differ between groups, although, as noted in Figure 2B, tumors over 1 mm^3^ were more frequent in the MCA/LMW PAH mixture/B[*a*]P group than in any other group. 

### 3.2. BALF Analysis in the Two-Stage Model Indicates Differences in Inflammatory Infiltrates Among Treatment Groups

**Figure 3:**
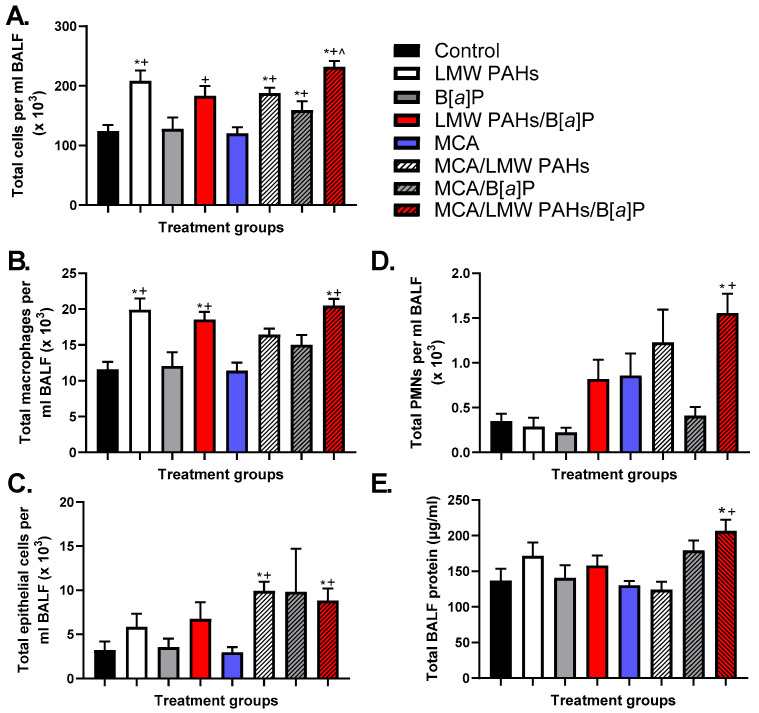
Bronchoalveolar lavage fluid (BALF) analysis of cellular infiltrates in BALB/ByJ mice 20 weeks following MCA reveals significant increases in total cells (**A**), macrophages (**B**), epithelial cells (**C**), PMNs (**D**), and total BALF protein concentration (**E**) in the MCA mice treated with the combined LMW PAH mixture and B[*a*]P as tumor promoters. Mean ± SEM presented. Control, n = 4; LMW PAH mixture, n = 8; B[*a*]P, n = 6; LMW PAH mixture + B[*a*]P, n = 10; MCA, n = 14; MCA + LMW PAH mixture, n = 8; MCA + B[*a*]P, n = 9; MCA +LMW PAH mixture + B[*a*]P, n = 14. *, *p* < 0.05 compared to the control treatment group; +, *p* < 0.05 compared to the MCA treatment group; ^ *p* < 0.05 compared to the MCA + B[*a*]P treatment group.

**Figure 4:**
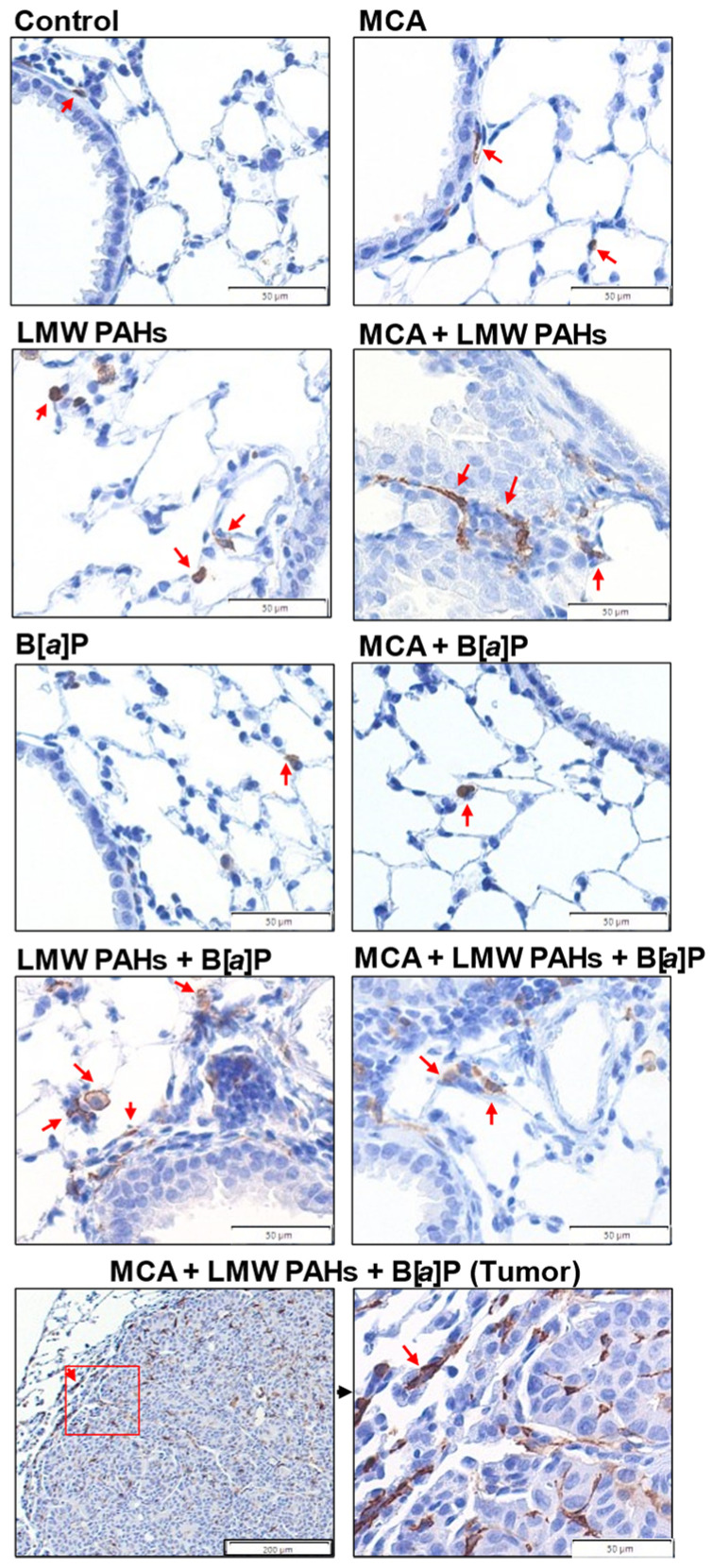
Immunostaining for macrophages demonstrates macrophages in the lungs with the LMW PAH mixture, similar to the BALF analysis for inflammatory cell infiltrates. F4/80 staining for macrophages in formalin-fixed inflated lungs on 5 μm sections; n = 3 mice per treatment. Red arrows indicate some of the macrophages, stained brown, but not all macrophages are noted. Images are at 40X magnification, except for the tumor (bottom image) at both 10 and 40X magnification. Bars indicate magnification.

Increases in total BALF cells were observed in several groups (Figure 3A), and this was primarily due to increases in macrophage populations (Figure 3B). The MCA/LMW PAH mixture/B[*a*]P group macrophage numbers were similar to those observed in most other LMW PAH mixture groups, while the B[*a*]P groups were not significantly different for any specific cell types. PMNs and epithelial cells were also significantly elevated (Figure 3C,D) only in the MCA/LMW PAH mixture/B[*a*]P group, further supporting the increased inflammatory response observed as well as increased epithelial sloughing in that treatment group. Additionally, the total protein content in the BALF was also significantly elevated only in the MCA/LMW PAH mixture/B[*a*]P group, further indicating increased lung hyperpermeability (Figure 3E). Immunostaining for both macrophages and PMNs also verified our results from the BALF analysis (Figure 4 and Figure S2), indicating more inflammatory infiltrates in the treatment group with significant tumor promotion (MCA/LMW PAH mixture/B[*a*]P). 

### 3.3. Secretion of KC and Increased mRNA Expression of Pro-Inflammatory Markers, Including a Bioactive Lipid Pathway

**Figure 5:**
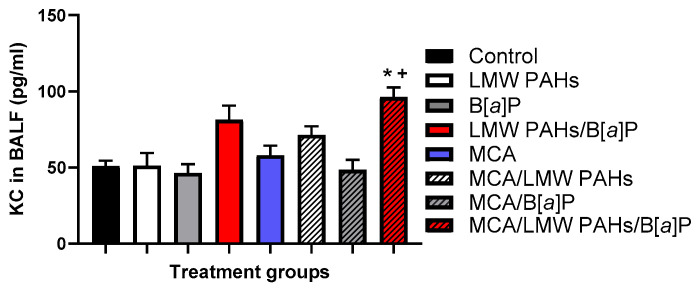
An increase in secreted BALF KC supports the lung inflammation in the MCA group combined with the LMW PAH mixture and B[*a*]P (red with line). LMW PAH mixture = LMW PAHs (1MeA:Flthn:Phe). Mean ± SEM presented. Control, n = 3; LMW PAH mixture, n = 6; B[*a*]P, n = 4; LMW PAH mixture + B[*a*]P, n = 9; MCA, n = 11; MCA + LMW PAH mixture, n = 8; MCA + B[*a*]P, n = 7; MCA +LMW PAH mixture + B[*a*]P, n = 13. *, *p* < 0.05 compared to the control treatment group; +, *p* < 0.05 compared to the MCA treatment group. No other groups were significant.

**Figure 6:**
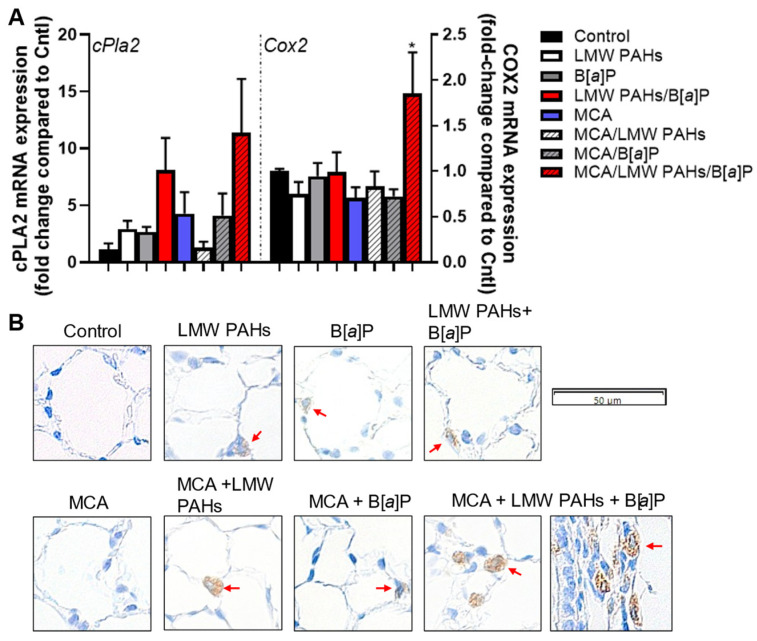
An eicosanoid pathway is elevated in response to these combinations of PAHs in the lung, specifically one pathway downstream of cPLA_2_, the cyclooxygenase (COX)2 pathway. (**A**) mRNA expression of cPLA2 trends towards an increase in the response to the combination of the MCA + LMW PAH mixture + B[*a*]P, and COX2 is significantly elevated in response to the same combination. Quantitative RT-PCR was performed on whole lungs from each treatment group; n = 3, control, MCA, and B[*a*]P, MCA + B[*a*]P; n = 4, LMW PAH mixture, LMW PAH mixture + B[*a*]P, MCA + LMW PAH mixture; n = 5, MCA + LMW PAH mixture + B[*a*]P. *, *p* < 0.05 compared to the control treatment group and every other group. (**B**) COX2 immunostaining in formalin-fixed lungs on 5 μm sections supports the mRNA expression observed. n = 3 mice per treatment. Red arrows indicate some of the positive brown-stained cells; not all are noted. Images are at 40X magnification; the bar indicates magnification.

KC, a chemokine often secreted from PMNs in order to recruit other cell types, was increased in the MCA/LMW PAH mixture/B[*a*]P treatment group (Figure 5). PMNs are often increased in the tumor microenvironment during the early stages of lung cancer development and are now under consideration as a potential indicator of worse outcomes for lung cancer patients [51,52,53]. *Mcp-1* gene expression was also increased in response to the same combined group, compared to all other groups, while other pro-inflammatory genes (*Il6* and *Il10*) were more variable (Figure S3A). Additionally, markers specific for macrophage populations such as *iNos*, *Arg1*, and *Cd206*, trended towards an increase in the MCA/LMW PAH mixture/B[*a*]P group (Figure S3B). Further, the pro-inflammatory bioactive lipid pathway (*Cox2*) was elevated in response to the MCA/LMW PAH mixture/B[*a*]P treatment group at the mRNA expression level for *Cox2* (Figure 6A), with a trend for increased *cPla2* gene expression. cPLA_2_ is upstream of the COX2 pathway [54]. We also observed increased COX2 staining in the lungs of the same treatment group (MCA/LMW PAH mixture/B[*a*]P) (Figure 6B).

### 3.4. Additional Pathways Involved in Several Other Markers for Early-Stage LUAD Considered Hallmarks of Cancer

**Figure 7:**
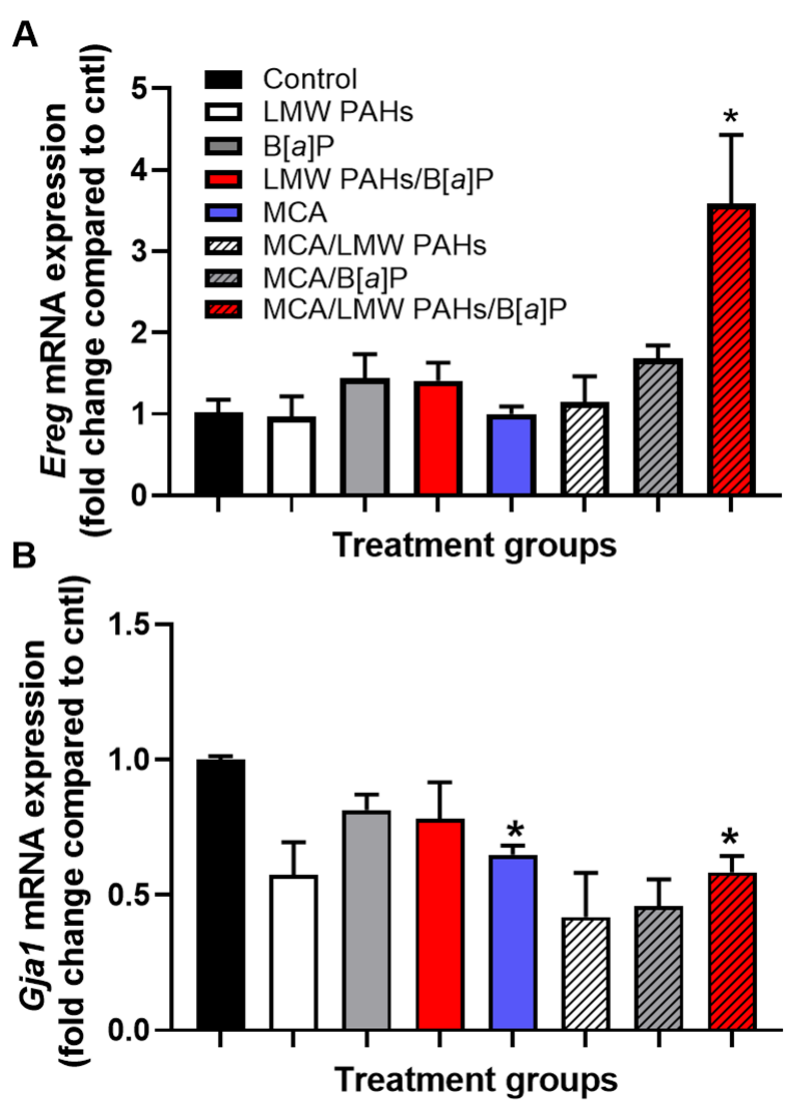
Two additional markers of specific hallmarks of cancer for lung by gene expression. (**A**) Epiregulin (*Ereg*) gene expression is significantly induced in response to the MCA/LMW PAH mixture/B[*a*]P treatment group above all other groups. (**B**) *Gja1*, the gene for connexin 43, is significantly reduced in response to the same treatment group, along with the MCA group. All groups with MCA have a trend towards reduced *Gja1* expression. For these studies, quantitative RT-PCR from whole lung tissues of treated mice was used to assess changes in *Ereg* and *Gja1*. Mean ± SEM presented. n = 3, control, MCA, and B[*a*]P, MCA + B[*a*]P; n = 4, LMW PAH mixture, LMW PAH mixture + B[*a*]P, MCA + LMW PAH mixture; n = 5, MCA + LMW PAH mixture + B[*a*]P. *, *p* < 0.05 compared to the control treatment group and all other groups unless noted.

There are several hallmarks of cancer that we have observed in our other in vivo and in vitro studies, two of which we provide supportive evidence here. One is epiregulin (EREG), a ligand for the EGFR receptor [55,56,57,58]; EGFR is a known pathway important to lung cancer development and involved in the proliferative hallmark of cancer. In a previous study, we demonstrated that Ereg was critical for lung tumor promotion in another two-stage model using *Ereg*-deficient mice on a BALB/ByJ background [44]. Further, increased EREG protein and mRNA are associated with a worse prognosis (overall survival) in patients with *KRAS* mutant LUAD [59,60]. We observed an increase in *Ereg* gene expression only in the MCA/LMW PAH mixture/B[*a*]P treatment group (Figure 7A). We also evaluated the hallmark of cancer related to the evasion of growth suppression by testing the gene expression of a specific gap junction gene called connexin 43 (*Gja1*), the most prevalent connexin in lung epithelial cells. This gene is often reduced in lung cancer and is linked to significantly worse overall survival for LUAD patients, according to our analysis using KMPLOT.com (Figure S4) [61]. We observed a significant reduction in *Gja1* expression in the MCA and MCA/LMW PAH mixture/B[*a*]P groups, but all groups with MCA were reduced. These additional markers are both supportive of our previous studies assessing these LMW PAHs in in vitro model systems [27,30,44].

## 4. Discussion

The WHO estimates that 20% of cancers worldwide are attributable to environmental risks, predominantly air pollution [62]. LUAD is the most prevalent form of lung cancer and the primary type of lung cancer that results from environmental and occupational exposures other than smoking cigarettes [3]. To formulate accurate assessments of risk and prevention of LUAD requires databases that integrate exposomes with the mechanistic effects of mixtures we are exposed to in cancer development. This study takes a significant step forward to address the issue of mixtures in LUAD using a two-stage lung carcinogenesis model. More specifically, we determined the in vivo tumorigenic effects of a mixture of LMW PAHs combined with the legacy HMW PAH, B[*a*]P, demonstrating significant increases in lung tumors along with mechanistic insights that include identifying BALF inflammatory infiltrates, pro-inflammatory transcripts, KC protein content, and mRNA expression of the gap junction (*Gja1*) and epiregulin (*Ereg*) genes.

Our fundamental understanding of LUAD and its premalignant lesions has increased due to advances in technology, yet the only current intervention strategy is to stop smoking [3]. Those patients with localized tumors have far higher 5-year survival than those with metastasized lesions (63.7 vs. 8.9%) [63], *thus more research on these early lesions and how and what pollutants cause these lesions is critical*. Unfortunately, increases in wildfires and temperatures both lead to increases in air pollutants, many of which contain the PAHs used in our studies [12,13,14]. Additionally, a strong percentage of the global population continues to smoke (~20%) [64]. Burn pits are an occupational example that contains these PAHs and expose our military and potentially civilians [15,21]. These other environmental and occupational exposures that contain PAHs (e.g., wildfires, secondhand smoke) are predicted to continue as a health threat to our population, which warrants more research [65,66]. While smoking rates have decreased in the U.S. as well as lung cancer overall, worldwide, lung cancer remains the number one cause of cancer mortality with ~1.8 million deaths/year and ~25% of cases due to non-smoking-related causes [67]. Further, we should not ignore exposures to PAHs from smoking. A re-evaluation of Philip Morris’ studies revealed that secondhand smoke components were more toxic and carcinogenic than mainstream smoke [68,69,70]. Several studies also showed significant levels of LMW PAHs in secondhand smoke of cigarettes and marijuana, with the greatest levels found in sidestream smoke [16,17]. For example, in Moir et al. (2008) [16], the PAH levels of one cigarette when computed over a year for a one-pack per day smoker (20 cigarettes/day) equates to levels that are comparable to those in our study (5.97 mg LMW PAH mixture/year and 176.7 μg B[*a*]P total per year) and demonstrate that these LMW PAHs are significantly higher (~33-fold) than the HMW species. This further illustrates the importance of understanding the adverse effects of human exposure to these more prevalent PAHs. Considering the exposure comparison noted here for these LMW PAHs in secondhand smoke in addition to other LMW PAHs not measured in this study, and along with other sources of PAH exposures such as air pollution (e.g., wildfires, etc.) and dietary ingestion, this can clearly create high exposure regimens.

This is the first study to assess the tumor-promoting potential of a mixture of specific LMW PAHs in a two-stage lung carcinogenesis model. Non-genotoxic LMW PAHs are presently not a major factor in contemporary risk assessment models on the carcinogenicity of PAHs. B[*a*]P is a known group 1 carcinogen; however, neither of the three LMW PAHs tested herein are considered carcinogens either by the IARC or the EPA. Our studies determined significant increases in lung tumor promotion only in those initiated mice exposed to both the LMW PAH mixture (Flthn, 1-MeA, and Phe) and B[*a*]P. The implication is that the LMW PAHs significantly increased the tumor-promoting properties of B[*a*]P, which was weak in the absence of the LMW PAHs. The LMW PAHs in the absence of B[*a*]P did not promote tumors. Thus, our data support that the selected LMW PAHs, namely, 1-methylanthracene, phenanthrene, and fluoranthene, are strong co-promoters of tumors. Other groups have observed primary lung tumors following exposure to B[*a*]P but at far higher concentrations (50 mg/kg by aspiration) [47]. In the future, studies are needed to test the individual LMW PAHs used here in combination with B[*a*]P to determine if the tumor promotion results from a specific PAH compound; however, we are not exposed to individual PAHs, but as PAH mixtures in the environment, and for some, in the workplace. In addition, the morphology differences between groups could also indicate a more aggressive tumor (i.e., papillary/mixed morphology) [71,72,73]; however, more studies are needed to expand the time frame for tumor development.

Next, we explored potential modes of action by investigating several hallmarks of cancer, namely, tumor-promoting inflammation (e.g., increased macrophages, PMNs, epithelial cells, KC, COX2) and proliferative responses (e.g., *Ereg*), as well as evasion of growth suppression (e.g., *Gja1*). Inflammation is a key cancer hallmark during the tumor promotion stage of lung cancer development, and the literature in this area is plentiful, including those focusing on specific macrophage phenotypes and PMNs in early lesions [51,52,53,74,75,76]. In our studies, the macrophages were found at the highest levels inside and surrounding the tumors (Figure 4), and the PMNs were highest surrounding the tumor but were not found within the tumor architecture (Figure S2), supporting the increased number of tumors in the MCA/LMW PAH mixture/B[*a*]P group. These results are consistent with our previous studies using similar models [43,44], including the increase in KC. While the mRNA expression of M1 (iNOS) and M2 (Cd206, Arg1) macrophage activation markers [43] was not significantly different, the analysis using whole lung RNA could reduce the signal in the macrophages. Future studies can test specific staining for these and other markers [77]. In addition, we have previously shown the involvement of pro-inflammatory bioactive lipid pathways, including increases in the pathways leading to cPLA_2_ cleavage of phospholipids upstream of COX2 that reside in the cell membrane, such as many phosphatidylcholines [30], and downstream of the COX2 pathway, such as PGE_2_, PGD_2_, and isoprostanes, in response to these same LMW PAHs (Flthn and 1-MeA) in mouse and human lung epithelial cells. A role for the COX2 pathway is well known for lung cancer, specifically LUAD, with numerous groups using COX2-specific inhibitors in the early 2000s until recently to reduce tumorigenesis, although they were relatively ineffective against LUAD [75,78,79,80,81]. 

Another hallmark is the increase in *Ereg* expression, a pathway linked to increases in proliferation, cell survival, and wound healing, among others [82]. EREG is a ligand for EGFR as well as ERBB4, which is in the same receptor family [55,56,57,58]. Using human and mouse lung epithelial cell lines [44], we observed that recombinant (r)EREG induced wound healing and increased cell growth at levels similar to EGF [44], thus supporting EREG as a marker of this hallmark for early-stage lung cancer. We and others also observed that EREG was critical to lung tumor development, either through an Ereg^−/−^ mouse model [44] or one with EREG over-production [83]. Our lung model exhibited significant increases in *Ereg* expression in only MCA-initiated animals treated with B[*a*]P plus the LMW PAH mixture but not in the absence of either alone. This finding supports that this combination of B[a]P plus the LMW PAH mixture links EREG to the actual tumor-promoting events and not to initiating events. Importantly, several studies have investigated human anti-EREG antibody development for treating diseases such as colon cancer [84] and, in the future, potentially as an intervention for lung cancer [82].

As noted above, the dysregulation of GJIC is a critical molecular event in the evasion of growth suppression, another hallmark of cancer [34]. The links between GJIC and inflammatory mediator responses to LMW PAHs (1-MeA and Flthn) were also previously determined by our group using inhibitors of P38 MAPK, cPLA_2_, and other anti-inflammatory inhibitors, where these inhibitors prevented the LMW PAH-induced dysregulation of GJIC in vitro [29,30]. As noted in Figure S4, higher mRNA expression of GJA1 (connexin 43) is significantly associated with higher overall survival in LUAD patients, specifically in stage 1 or early-stage lung cancer. Further, *Gja1^−/+^* (Cx43) mice also have significantly increased lung tumor susceptibility [36,85]. Thus, this marker in our model is critical for early-stage lung cancer, and additional studies are needed to investigate potential targets to reverse the inhibition of GJA1 function observed in response to the MCA/LMW PAH mixture/B[*a*]P treatment.

Lastly, we acknowledge that there are limitations of this study and discuss them here. This was not a comprehensive study of mixtures, including mixtures of PAHs, but rather a significant step forward to begin the carcinogenic assessment of PAH mixtures containing a subset of environmentally relevant LMW PAHs, a highly overlooked class of PAHs due to their non-genotoxic potential. We acknowledge the use of a higher dose for our first study to ensure a robust response to the PAH mixtures. Our results can now be used for a follow-up study with lower doses, where we predict that LMW PAHs will significantly increase B[*a*]P-induced tumors. We also examined only one time point. Earlier time points might reveal additional differences between the treatment groups, such as early proliferation and inflammation pathways, to better understand the tumor-promoting events. Additionally, extending the time to longer periods, such as 24–30 weeks, as we have done in the past with other promoters [86,87], could also be important to determine later events in the sequence of lung tumor development and any additional morphological differences in tumors. Using micro-CT is also something we plan to use for future studies to follow early lesions throughout development; however, the COVID-19 pandemic made it impossible to examine these mice using micro-CT at our university. Any sex differences were not determined with this study using only female mice. There is evidence of sex differences in lung cancer [88]. Further, we did not perform a comprehensive evaluation of immune cell infiltration; however, in the future, we plan to assess spatial immunostaining and spatial transcriptomics in our samples to understand the full extent of the differences in responses to the combination of LMW PAHs and B[*a*]P as tumor promoters.

## 5. Conclusions

Traditionally, PAH cancer studies have focused on individual PAHs, which can greatly underestimate their contributions to cancer [38,39,40]. Further, most of the LMW PAHs have been greatly overlooked due to their lack of genotoxicity, with the exception of naphthalene [20]. Our results with mice treated with environmentally prevalent LMW PAHs support that these LMW PAHs in combination with B[*a*]P, and potentially other environmentally relevant LMW PAHs, can have major effects on adverse health outcomes concerning cancers, at least LUAD, which can be missed with studies focused on individual PAHs. To ensure accurate assessments of cancer risks and likely other risks of adverse health outcomes, there is a need to conduct more research on PAH mixtures and to not exclude the non-genotoxic PAHs. This concept of including non-genotoxic compounds in cancer studies would also apply more broadly to other environmental mixtures.

## Data Availability

The original contributions presented in this study are included in this article/Supplementary Material. Further inquiries can be directed to the corresponding authors.

## Supplementary Materials

The following are available online at https://www.mdpi.com/article/10.3390/toxics12120882/s1, Supplementary Figures S1–S4. Figure S1: Histology for all treatment groups 20 weeks following MCA. Figure S2: Immunostaining for PMNs in the lungs demonstrates PMNs in those lungs with LMW PAHs, similar to the BALF analysis for inflammatory cell infiltrates. Figure S3: Pro-inflammatory markers as well as macrophage activation markers in response to the combined PAHs in the two-stage model. Figure S4: KMPLOT.com analysis for the GJA1 gene in lung adenocarcinoma patients showing that higher GJA1 mRNA expression significantly associates with overall survival in lung adenocarcinoma.
