# Supplementary material for: Non-Genotoxic and Environmentally Relevant Lower Molecular Weight Polycyclic Aromatic Hydrocarbons Significantly Increase Tumorigenicity of Benzo[a]pyrene in a Lung Two-Stage Mouse Model
Source: Toxics. 2024 Dec 2;12(12):882. doi: 10.3390/toxics12120882 (PMC11679119; doi:10.3390/toxics12120882)
Supplement: Supplementary file 1 [file toxics-12-00882-s001.zip › toxics-3313588-supplementary.pdf]

Non-genotoxic and environmentally relevant lower molecular weight polycyclic aromatic hydrocarbons significantly increase tumorigenicity of benzo[*a*]pyrene in a lung two-stage mouse model

Supplemental figures

A.K. Bauer et al.

2024

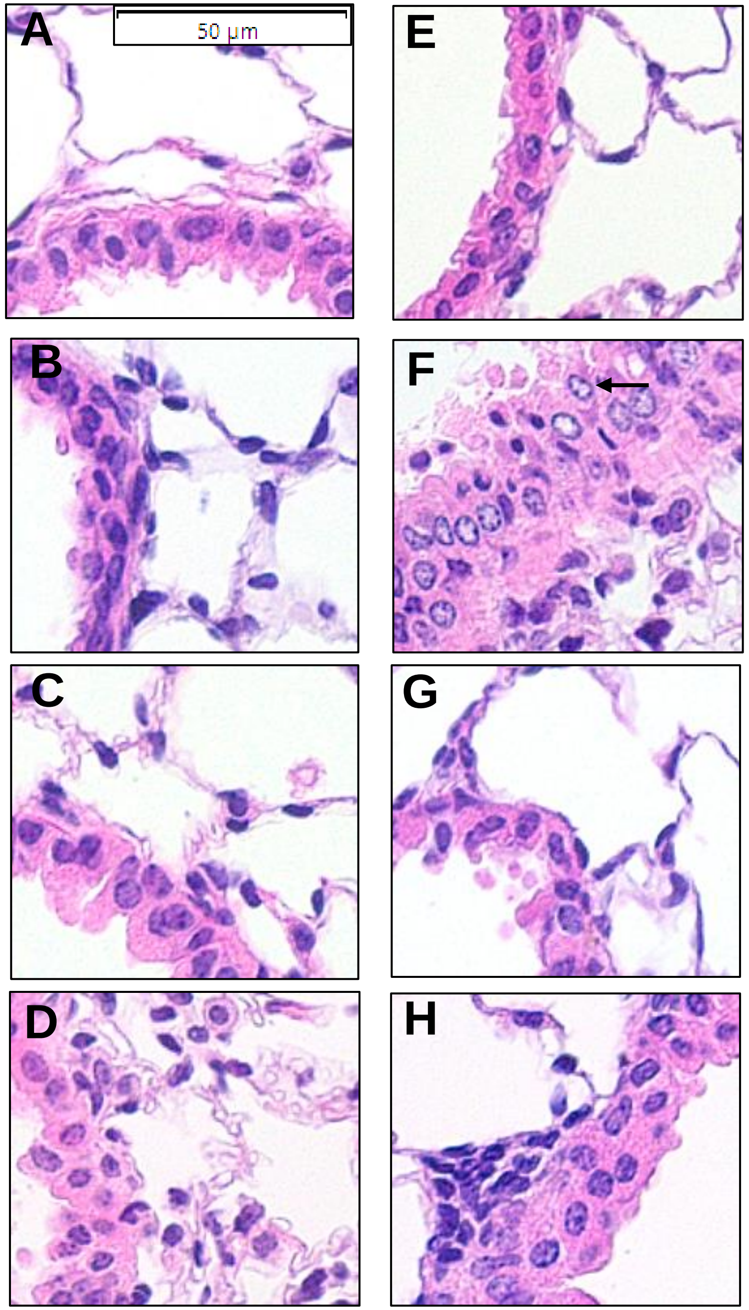

Figure S1: Histology for all treatment groups 20 weeks following MCA. (A) Control; (B) LMW PAH mixture; (C) B[*a*]P; (D) LMW PAH mixture + B[*a*]P; (E) MCA; (F) MCA + LMW PAH mixture; (G) MCA + B[*a*]P; (H) MCA + LMW PAH mixture + B[*a*]P. An increase in cellularity as well as a thickening in the airways and airspaces are observed in B, F, C, D, and H. Nuclear atypia is also present in some areas in F (black arrow).

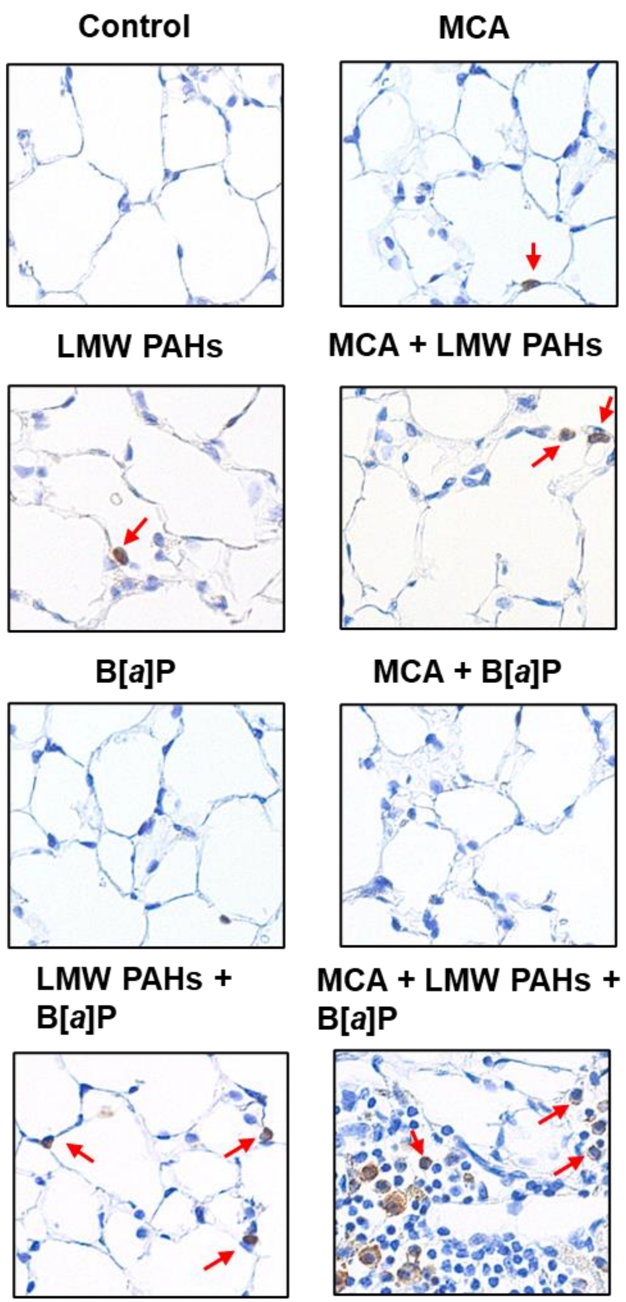

Figure S2: Immunostaining for PMNs in the lungs demonstrates PMNs in those lungs with LMW PAHs, similar to the BALF analysis for inflammatory cell infiltrates. NIMP-R14 staining for PMNs in formalin-fixed inflated lungs on 5 μm sections; n=3 mice per treatment. Red arrows indicate some of the PMNs, stained brown, but not all at noted. Images are at 40X magnification. Bars indicate magnification.

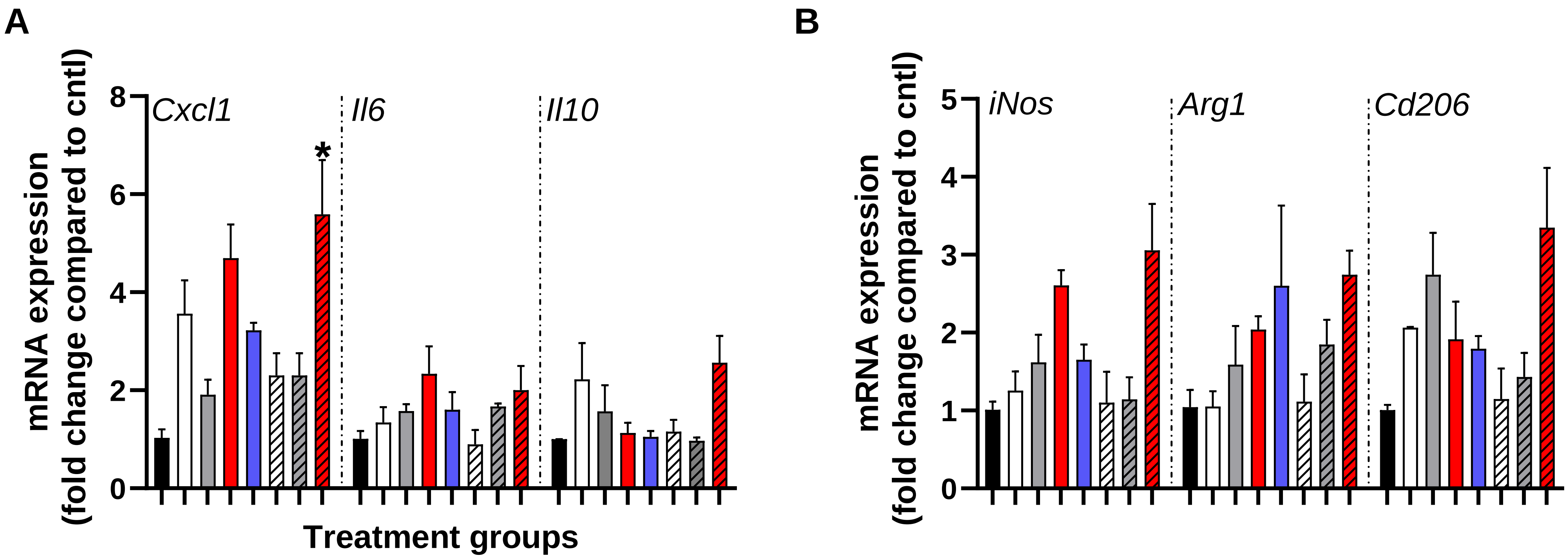

Figure S3: Pro-inflammatory markers as well as macrophage activation markers in response to the combined PAHs in the two-stage model. A) mRNA expression of Cxc1 is significantly elevated in response to the combination of MCA + LMW PAHs + B[*a*]P, while Il6 and Il10 are unchanged. B) iNOS and CD206 macrophage activation markers trend towards an increase in the combination of MCA + LMW PAHs + B[*a*]P, but not significantly. Quantitative RT-PCR was done on lung from each treatment group; n=3, control, MCA, and B[*a*]P, MCA + B[*a*]P; n=4, LMW PAHs, LMW PAHs + B[*a*]P, MCA + LMW PAHs; n=5, MCA + LMW PAHs + B[*a*]P. *, $p<0.05$ compared to control treatment group and every other group.

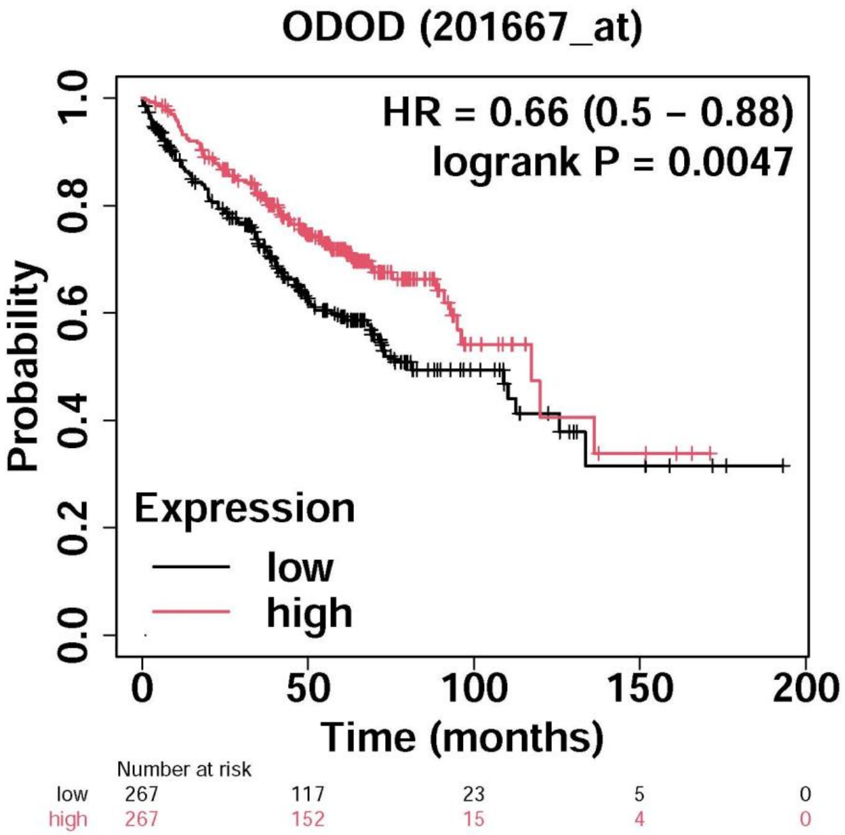

Figure S4: KMPLOT.com analysis for the *GJA1* gene in LUAD patients showing that higher *GJA1* mRNA expression significantly associates with overall survival in LUAD. Multivariate analysis was performed (co-variates: gender and stage) and N= 534 patients; analyzed via kmplot.com. Hazard ratio (HR), 95% confidence intervals, and logrank P presented. In addition, when analyzed for only stage 1 LUAD patients, higher *GJA1* mRNA expression associates with increased overall survival (HR=0.63, logrankP=0.019, n=370 patients).
